# First Intraoperative Radiation Therapy Center in Africa: First 2 Years in Operation, Including COVID-19 Experiences

**DOI:** 10.1200/GO.20.00258

**Published:** 2020-11-06

**Authors:** Yastira Ramdas, Carol-Ann Benn, Michelle van Heerden

**Affiliations:** ^1^ Breast Care Unit, Netcare Milpark Hospital, Johannesburg, South Africa; ^2^ Netcare Medical Physics CoE, Netcare Limited, Sandton, South Africa

## Abstract

**PURPOSE:**

There is a shortage of radiation therapy service centers in low- to middle-income countries. TARGIT–intraoperative radiation therapy (IORT) may offer a viable alternative to improve radiation treatment efficiency and alleviate hospital patient loads. The Breast Care Unit in Johannesburg became the first facility in Africa to offer TARGIT-IORT, and the purpose of this study was to present a retrospective review of patients receiving IORT at this center between November 2017 and May 2020.

**PATIENTS AND METHODS:**

Patient selection criteria were based mainly on the latest American Society of Radiation Oncology guidelines. Selection criteria included early-stage breast carcinoma (luminal A) and luminal B with negative upfront sentinel lymph node biopsy that negated external-beam radiation therapy (EBRT). Patient characteristics, reasons for choosing IORT, histology, and use of oncoplastic surgery that resulted in complications were recorded.

**RESULTS:**

One hundred seven patients successfully received IORT/TARGIT-IORT. Mean age was 60.8 years (standard deviation, 9.3 years). A total of 73.8% of patients presented with luminal A, 15.0% with luminal B, and 5.6% with triple-negative cancer. One patient who presented with locally advanced breast cancer (T4N2) opted for IORT as a boost in addition to planned EBRT. Eighty-seven patients underwent wide local excision (WLE) with mastopexy, and 12 underwent WLE with parenchymal. Primary reasons for selecting IORT/TARGIT-IORT were distance from the hospital (43.9%), choice (40.2%), and age (10.3%).

**CONCLUSION:**

This retrospective study of IORT/TARGIT-IORT performed in Africa confirms its viability, with low complication rates and no detrimental effects with breast conservation, resulting in positive acceptance and the potential to reduce Oncology Center patient loads. Limitations of the study include the fact that only short-term data on local recurrence were available. Health and socioeconomic value models must still be addressed in the African setting.

## INTRODUCTION

Breast cancer is one of the most common types of cancers affecting women, second to skin cancer, with a 100 times greater prevalence rate in women than in men.^1^ Early detection of breast cancer, coupled with radiation treatment, can dramatically reduce mortality rates and reduce the need for mastectomies.^2^ Alternatives to a mastectomy are breast conservation surgery (BCS) followed by a 6- or 7-week course of external-beam radiation therapy (EBRT).^3^ The postoperative radiation therapy (RT) regimen, if not completed, adversely affects the efficacy of treatment and increases the need for a mastectomy. Delivering a single fraction using TARGIT–intraoperative radiation therapy (IORT) (ClinicalTrials.gov NCT00983684) in theater after surgery reduces the need for postsurgical treatment, reduces the impact on the patient’s lifestyle, and reduces cost.^3^

**CONTEXT**

**Key Objective**
What are the results and nuances observed for patients treated at the first intraoperative radiation therapy (IORT) facility in Africa?
**Knowledge Generated**
This retrospective study of IORT performed from November 2017 to May 2020 at the first IORT treating facility in Africa confirms its viability, with low complication rates (2.8%, in line with other published research), low recurrence rates (1.9%, two cases of 107), and no detrimental effects on breast conservation. No TARGIT–IORT recurrences were recorded in this study, but two IORT recurrences (ductal carcinoma in situ group) were noted.
**Relevance**
TARGIT-IORT can be a tantalizing option for patients meeting the criteria who are reluctant to travel long distances, would like to avoid numerous trips to medical facilities, and are averse to frequent cycles of external-beam radiation therapy.

Although numerous studies detail the efficacy of IORT in early-stage breast carcinomas, limited information is available characterizing the post-treatment affects or detailing any nuances within the Southern Africa patient context.^4^ Traditional long-term studies deal primarily with recurrence rate, survival tests, and efficacy comparisons with EBRT.

TARGIT-IORT has been largely adopted in developed countries, with protocols based on these countries; however, there is a paucity of data of value in middle- and lower-income countries such as South Africa. Exacerbating this problem was the complete absence of operational TARGIT-IORT equipment in Africa before November 2017.

The emergence of a disease presenting with primary acute viral respiratory tract infection and other severe symptoms, which emanated from Wuhan, China, in late 2019, named COVID-19 and caused by SARS-CoV-2 infection, has resulted in a global epidemic spreading across all habitable regions.^5,6^ This has resulted in the implementation of extreme procedural measures to limit viral transmission in hospitals. However, improved cleaning procedures have led to reduced operating capacity in oncologic centers.^7,8^ Factors negatively affecting radiation treatment adherence include the requirement for COVID-19 tests before treatment, a lack of COVID-19 testing kits, the length of time it takes to receive COVID-19 test results, social distancing measures, and forced isolation.

The purpose of this study was to characterize patient demographics; record multifactorial post-treatment effects such as complications, both local and those related to cardiac response; and identify early and late toxicities in adult females diagnosed with early-stage breast cancer receiving IORT/TARGIT-IORT in South Africa from November 2017 to May 2020. Subpopulation group benefits, quality of life, experiences with COVID-19 hospital protocols, and the value added by shorter treatment times compared with longer traditional EBRT procedures were also documented.

## PATIENTS AND METHODS

### Study Design and Ethics Approval

A retrospective analysis was performed on data collected from the Netcare Milpark Breast Care Unit from November 2017 to May 2020. The study investigated clinical outcomes and toxicity/adverse effects/complications associated with 107 consecutive patients (n = 107). Each patient signed an informed consent, and institutional ethics approval was obtained from Pharma-Ethics^9^, with ethics reference number (Protocol number) 180520207.

### Study Population, Selection, and Methods

The study population consisted of adult females displaying symptoms congruent with early-stage breast carcinoma who presented at the study site between November 2017 and May 2020. Patients presented primarily from the Southern African region.

Before considering TARGIT-IORT for a patient presenting with early-stage breast cancer (typically, luminal A type) detected by imaging or examination, a confirmation sentinel lymph node biopsy was performed to confirm negative lymph nodes together with other relevant factors. Patients were discussed in a multidisciplinary meeting before being assessed by specialist surgeons and radiation oncologists, who discussed applicable treatments, including advantages, disadvantages, and funding; if TARGIT-IORT was selected, informed consent was obtained. Patients choosing alternative therapies were referred to appropriate treatment facilities.

Inclusion criteria, which were more restrictive than those in the TARGIT-A trial, included patients presenting between November 2017 and May 2020 who had medical records available for review and those mainly meeting American Society of Radiation Oncology (ASTRO) guidelines.^10^ Notably, patients who were included were those who were older than 50 years and who met the following criteria: invasive ductal carcinoma, tumor stage T1/T2, and tumor size < 3.5 cm; ductal carcinoma in situ (DCIS) was included if it was low to intermediate grade and < 2.5 cm, with resection margins > 1 cm.^10^ Exclusions included bilateral breast cancer at the time of diagnosis. However, any severe concomitant disease that may have limited life expectancy or a history of malignant disease did not preclude entry if the expectation of relapse-free survival at 10 years was 90% or greater.

The majority of patients selected for TARGIT-IORT presented with early-stage disease and had a negative upfront sentinel lymph node biopsy, negating the need for EBRT. Patient characteristics, histology, the use of oncoplastic surgery, and any early complications were recorded.

**FIG 1:**
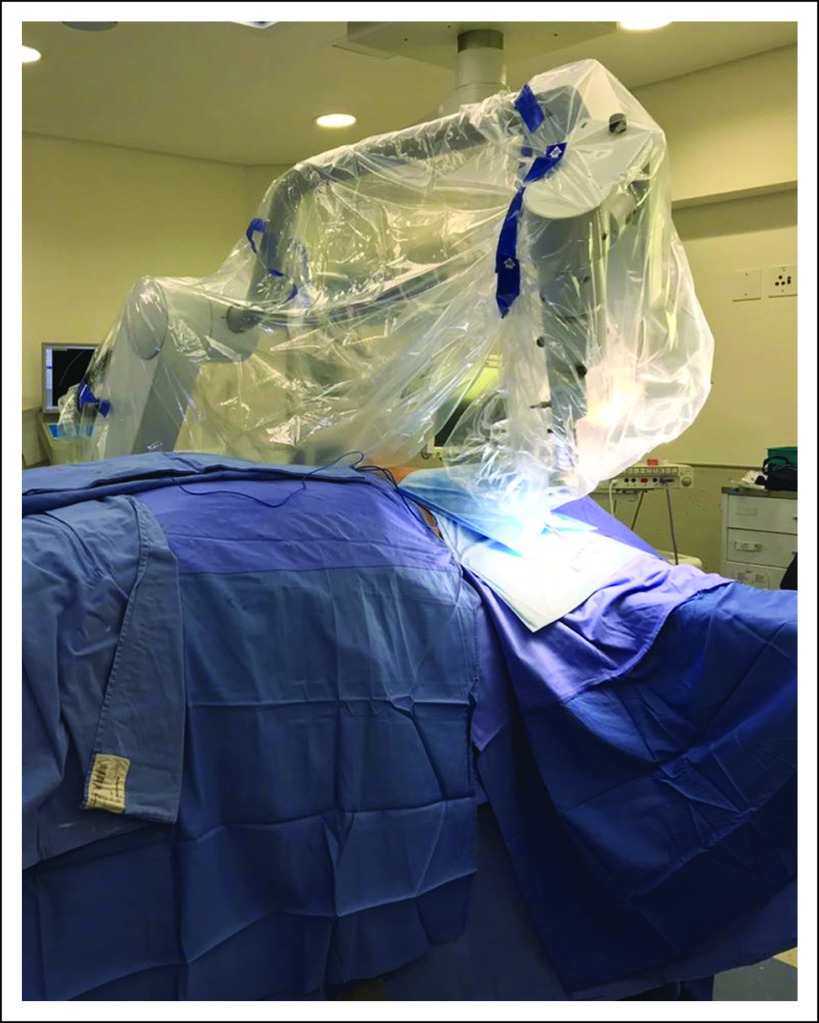
IORT Intrabeam 50kV, Carl Zeiss Surgical, Oberkochen, Germany located in Breast Care Unit in Netcare Milpark, Johannesburg.

**FIG 2:**
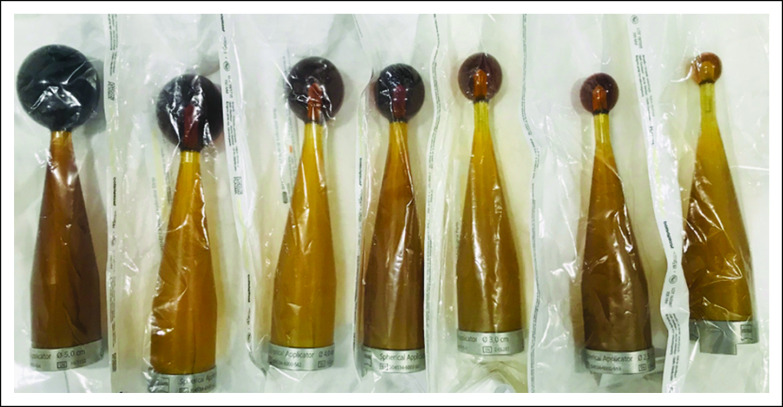
IORT Intrabeam 50kV, Carl Zeiss Surgical, Oberkochen, Germany applicators ball types.

BCS with sentinel node harvesting was took place, after which TARGIT-IORT was performed using the Intrabeam system (50 kVp, Carl Zeiss Surgical, Oberkochen, Germany), as presented in Figure 1. After lumpectomy, spherical treatment applicators, 5, 4.5, 4, and 3.5 cm in size, used routinely, as shown in Figure 2, were attached to the probe-like end of the unit, surgically inserted in the tumor bed, and closed via a purse-string suture, ensuring close apposition to the target tissues and reducing stray radiation to unintended tissue.

Before each clinical case, the output of the Intrabeam mini-accelerator x-ray source (XRS) was measured using a PTW 23342 ionization chamber (IC) together with the probe adjuster/IC holder quality assurance device. The measured exposure was converted to absorbed dose to water using a factor of f = 0.881 cGy/Roentgen. Typically, a 20-Gy radiation dose at 0 mm depth is delivered to the tumor bed, with treatment times in the range of 16 to 50 min determined using the manufacturer’s calibration depth-dose curves. Iso-dose curves were verified using a special water phantom device provided by the manufacturer with a PTW-34013 soft chamber. This allowed the XRS to be mounted superiorly on the top of the phantom device with the probe intercepting the water bath; this probe could be adjusted incrementally in any three-dimensional (x, y, z) direction.

## RESULTS

One hundred seven patients were selected to undergo TARGIT-IORT during lumpectomy at the Breast Care Center between November 2017 and May 2020, with an average 2-year follow-up period per patient.

The mean age was 60.8 years (standard deviation, 9.3 years), and all patients were female. The patient and tumor characteristics are listed in Table 1. Coincidental tumors were not reported. No telangiectasia was noted for any patients in this study.

**TABLE 1:**
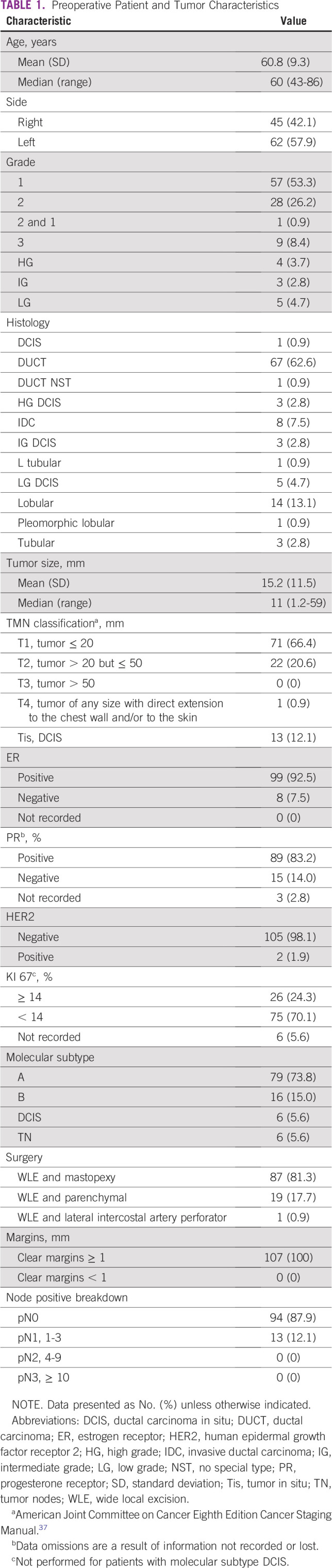
Preoperative Patient and Tumor Characteristics

### Adoption of IORT/TARGIT-IORT and After Treatment

Before administering the TARGIT-IORT treatment regimen, distance (43.9%) and choice (40.2%) were reported as the patients’ indication and primary reasons for opting to undergo TARGIT-IORT (Table 2). Patients were more willing to choose IORT/TARGIT-IORT because of delayed treatment times at EBRT machines, potential contact at hospital treating facilities, and general unease during the COVID-19 period. A recurrence rate of 1.9% (two of 107) was reported. A complication rate of 2.8% (three of 107), defined as major complications (fat necrosis and/or skin erythema), was reported.

**TABLE 2:**
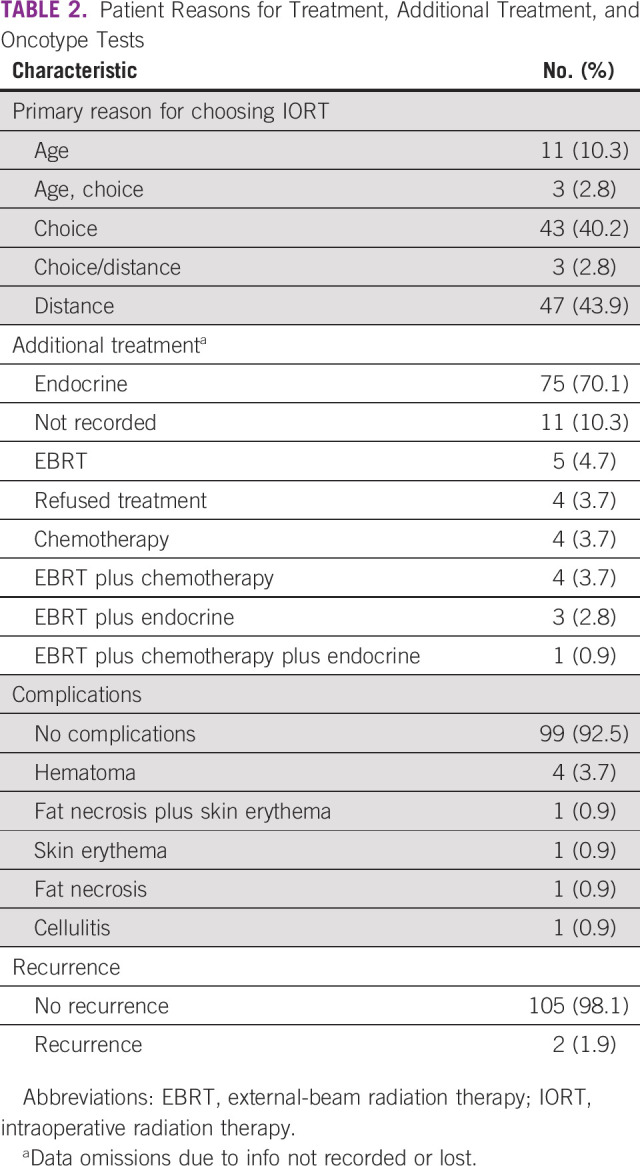
Patient Reasons for Treatment, Additional Treatment, and Oncotype Tests

### COVID-19 and IORT Procedure

The first confirmed case of COVID-19 in South Africa was reported on March 1, 2020, with a complete nationwide lockdown implemented on March 26, 2020. Five patients were treated between December 2019 and May 2020. No alterations to the TARGIT-IORT protocol were undertaken during this period. However, before surgery, patients suspected of contracting COVID-19 had to test negative to limit exposure to clinical staff.

## DISCUSSION

In recent history, breast cancer mortality rates among developed countries have dropped, with the United States recording a 39% decreased mortality rate in breast cancer from 1989 to 2015.^11^ Conversely, a greater rise in the incidence of breast cancer was reported in developing countries (883,000) than in developed countries (794,000) in 2018.^12^

IORT is a combined modality therapy used to treat breast cancer by administering radiation directly to the tumor bed after surgery.^13^ IORT has gained popularity in the past two decades, with multiple studies, including outcome-based studies, detailing its efficacy and noninferior results as compared with EBRT for early-stage breast cancer.^10,14-21^ IORT as a single fraction immediately after surgery (partial mastectomy) for early-stage small breast cancers, categorized as accelerated partial breast irradiation, has shown to produce positive cosmetic results with a single fraction of in situ RT delivered intraoperatively.^22^ More than 90% of recurrences of breast cancer occur at or near the tumor bed; hence, a targeted dose of radiation near the operation site should intuitively reduce the recurrence rate.^20,23,24^

Studies have revealed low local recurrence rates in standard-risk patients who received TARGIT-IORT rather than EBRT.^3,17,21^ The current study showed a 1.9% (two of 107) recurrence rate. In both of these cases, the patients had high grade (HG) and DCIS characteristics, suggesting that the cancer growth pattern type with HG paired with DCIS is not managed adequately with IORT and that EBRT should be offered instead. Additional studies with a longer follow-up should be undertaken to ascertain the causes and mechanisms associated with HG-DCIS recurrence rates. Although patients presenting with HG-DCIS were not included in the TARGIT-A trial, preliminary research provided evidence that TARGIT is a viable option for patients with DCIS because a low risk of additional therapy (7.3%) and a local recurrence rate of only 4.3% were reported.^25^

The Intraoperative Radiotherapy With Electrons (ELIOT) trials assessed the frequency and grade of RT–induced pulmonary fibrosis, comparing patients receiving intraoperative electron radiation therapy (IOERT) with those receiving EBRT; the results showed that 4% of the ELIOT arm versus 46% of the EBRT arm developed pulmonary fibrosis, with a higher risk-adjusted recurrence rate in the EBRT arm.^14^ Although this study provides compelling evidence, notable differences from contemporary IORT studies included the use of IOERT equipment and the visual assessment used for the evaluation of fibrosis.

A recent retrospective study of 127 female patients from 2009 to 2016 characterized patients treated exclusively or with TARGIT-IORT (Zeiss Intrabeam) and detailed demographics, treatment outcomes, complication rates and the feasibility of IORT as a substitute for salvage mastectomy.^14^ Limitations of the study included the use of a cohort restricted to a single institution with a short follow-up time (4 years) as compared with the minimum 5 years recommended.

The TARGIT-A randomized trial, the largest noninferiority trial with 33 centers in 11 countries, tested the risk-adjusted RT of breast carcinomas between TARGIT-IORT and EBRT while reporting local occurrence rates and overall survival.^17^ The study consisted of 3,451 patients (1,721 patients randomly assigned to a TARGIT-A group and 1,730 randomly assigned to an EBRT group), and it reported noninferior 5-year risk rates for local recurrence in conserved breast treatment of TARGIT-IORT verses EBRT and lower overall mortality for TARGIT-IORT (3.9% [95% CI. 2.7% to 5.8%]) compared with EBRT (5.3% [95% CI. 3.9% to 7.3%]).^26^ The study concluded that TARGIT-IORT was noninferior to EBRT and was a viable alternative for patients meeting the criteria, laying a scientific foundation for other efficacy trials comparing traditional cancer therapies.^17^ These findings were reaffirmed by the updated study and recommended TARGIT-IORT for breast carcinomas in patients ≥ 45 years of age with hormone-sensitive invasive ductal carcinoma up to 3.5 cm in size.^27^

Treatment with the Intrabeam system has been shown to be safe and effective^3,21,27,28^; however, it has been reported that the system requires improvement in the accuracy of the delivered dose.^29-31^ The calibration method, which was established before the start of the TARGIT-A trial (before the year 2000), has been maintained to ensure consistency of the delivered prescription dose, even though newer dosimetry protocols for photons in the kilovoltage energy range have been developed.^30,32,33^

Patients presenting at this study site displayed unique demographic characteristics and traveled long distances from African countries. Implementation rates of TARGIT-IORT therapies among clinicians in South Africa are low because of a lack of awareness, limited TARGIT-IORT expertise, and the complete absence of other facilities in Africa. These low adoption rates are mirrored by local medical aid funds, which results in most patients funding treatment modalities out of pocket. These factors, driven by the low socioeconomic status of the Southern African region, together with logistics, treatment funding, stigmatization of cancer, and other nonmedical-related taboos, adversely affect treatment adherence.

Because of low medical aid acceptance rates, the majority of patients (> 90%) paid for their treatment, which consisted of surgical, hospital, oncology, technical, and professional fees, using their own resources, which resulted in TARGIT-IORT/IORT being available for the wealthy only. Post-treatment follow-up has yet to record a single case of post-decision dissonance related to IORT/TARGIT-IORT, and most patients recommended the procedure to peers.

A low complication rate of 2.8% (three of 107) was noted, and this compared well with that reported by the TARGIT-A trial of 3.3% when using a similar definition of complication rate.^25^ Four patients experienced hematomas, each requiring a single guided aspiration, and there was no subsequent accumulation of fluid. One patient experienced fat necrosis, but this softened up gradually over 1 year. One patient reported skin erythema, which was treated and resolved with steroid cream. One patient had both skin erythema and fat necrosis; the former was treated with steroid cream and the latter soften gradually over 1 year. The fourth patient reported cellulitis that could not be linked definitively to IORT exposure.

The outbreak of COVID-19 adversely affected the South African health sector through the enforcement of extreme procedural changes specifically in oncologic centers, such as deep cleaning in between patient treatments, social distancing measures, 14-day quarantine rules for those testing positive for COVID-19, and timed slots that reduced the number of radiation treatments per day. Recent discussions within the radiation oncology community have highlighted the challenges affecting patient treatment, such as the patient’s frailty because of advanced disease, colocation with other patients in waiting areas, the specialized staff skill set being negatively affected, interruptions in treatments, and immune-compromised patients and palliative treatments being deprioritized.^34^ These challenges pose notable problems for patients requiring RT, such as the need to present numerous times at hospital treatment centers over a 6-week period. In particular, those meeting the TARGIT-IORT criteria are both elderly and immune compromised, which are key risk factors associated with the disease.^5^ Since December 2019, patients have become more amenable to IORT, because of single treatment therapy in surgery, minimized total hospital contact, and reduced risk of poor treatment adherence.

To the best of our knowledge, before and during the study period, no other research center in Africa was able to administer TARGIT-IORT. This was determined by a comprehensive literature review, media search, verification with major manufacturers such as Carl Zeiss, international IORT conference review, and confirmation with the TARGIT-A trial team in the United Kingdom.^31^

Thirteen patients received EBRT after IORT treatment, either exclusively or in combination with endocrine therapy and/or chemotherapy. Three patients who received preoperative chemotherapy were meant to continue exclusively with EBRT but opted for IORT in combination with EBRT to reduce treatment times because they lived in rural Africa, travel was a problem, and extending treatment time was not an option. Ten patients had upstage of histology, including positive lymph nodes or human epidermal growth factor receptor 2–positive and higher oncotypes, which migrated them out of the TARGIT-A recommended guidelines hence, they required EBRT.

As an estimate, more than 60% patients who presented at the study site with histologies similar to the prospective cohort before inception of TARGIT-IORT elected to have BCS. All treatment options, which included mastectomy and BCS, were presented to patients, but the majority elected breast conservation.

TARGIT-IORT has been proven to be noninferior to traditional EBRT treatments, and subsequent research is underway to further hone its applicability, protocols, and benefits.^17,26,27^ Simultaneously, it is predicted that the number of new cancer cases will increase, with those in the United States projected to increase to 2.3 million (a 45% increase) by 2030.^35^ Factors negatively affecting newly diagnosed patients receiving adequate cancer care include a nonuniform oncology practice location, a shortage of oncologists, and lack of access to quality care.^35^ In this study, a critical indication for patients opting for IORT/TARGIT-IORT was distance (43.9%), although the actual travel distance from home to the radiation center was not recorded because multiple factors play a role in the concept of distance in the African environment. Patients from other sub-Saharan countries have limited access to radiation machines; they form a geographically diverse patient base with many from nonurban districts forced to travel long distances to attend radiation centers; and those in poorer socioeconomic areas lacking a regular domicile address have the added burden of transport logistics requiring various and multiple public transport services.^134,36^ Patients who chose TARGIT-IORT can expect to travel significantly fewer kilometers related to RT and contribute to lower carbon dioxide emissions which was reported for patients, treated in the UK, forming part of TARGIT-A trial measuring less distance traveled for treatment as compared with those undergoing EBRT.^36^ This retrospective cohort study took place within a 5-year period, which is insufficient time to draw meaningful conclusions related to local recurrence related and time to systemic progression; hence, additional follow-up is required. No TARGIT-IORT recurrences were recorded in this study; however, two IORT recurrences (DCIS group) were noted.

TARGIT-IORT can be a tantalizing option for patients meeting the criteria who are reluctant to travel long distances, would like to avoid numerous trips to medical facilities, and are averse to the frequent cycles of EBRT. However, patients in the elderly subgroup (> 65 years) have reported difficulty in securing daily transport to and from oncology treatment centers because of reliance on family members, lack of adequate public transport, and lack of disposable income for private hire. Follow-on studies should investigate the long-term follow-up of the TARGIT-IORT patient group, potentially comparing this cohort with a cohort composed of EBRT patients, comparing factors such as complication rate, quality of life, and patient treatment preferences (mastectomy, etc).

This retrospective study of IORT performed in Africa confirms its viability, with low complication rates and no detrimental effects on breast conservation, resulting in positive acceptance and the potential to reduce Oncology Center patient loads caused by the COVID-19 pandemic. Limitations of the study include the use of only short-term data on local recurrence. Health and socioeconomic value models must still be investigated in the African setting.
